# The association between family capital and physical exercise among young and middle-aged adults: the potential mediating pathway of intergenerational transmission of education

**DOI:** 10.3389/fpubh.2026.1791251

**Published:** 2026-05-08

**Authors:** Fan Yang, Qiang Yi, Yi Yang, Ling Wang

**Affiliations:** 1 Department of Physical Education, Sichuan International Studies University, Chongqing, China; 2 College of Physical Education and Health, Chongqing Three Gorges College, Wanzhou, China; 3 College of Liberal Studies, Chongqing Three Gorges Vocational College, Wanzhou, China

**Keywords:** CGSS, family capital, intergenerational transmission of education, physical exercise, potential mediating pathway

## Abstract

**Introduction:**

Against a backdrop of increasing attention to health inequities, behavioral differences in physical exercise among young and middle-aged adults should be examined through structural factors such as family capital and the intergenerational transmission of education.

**Methods:**

This study uses data from the 2023 China General Social Survey and selects a sample of 2,394 adults aged 18–60. An ordered probit model tests the statistical association between family capital and exercise frequency, the bootstrap method verifies the indirect association of intergenerational educational transmission, and subgroup regressions combined with Suest tests analyze heterogeneity across urban/rural residence, gender, and income groups.

**Results:**

(1) both economic and cultural capital are significantly positively associated with physical exercise, with the association of cultural capital being more robust; social capital does not show a significant direct association; (2) offspring’s education plays a significant partial mediating role between family capital and physical exercise, and the mediation proportion is higher for cultural capital than for economic capital; (3) heterogeneity analysis indicates that the association of economic capital is stronger in rural and low-income groups, whereas the association of cultural capital is more pronounced among urban residents and women, which may reflect the deep mechanism through which cultural capital shapes health behaviors via habitus.

**Discussion:**

Physical exercise behavior exhibits clear intergenerational transmission, and education is the core pathway through which family capital is associated with offspring’s healthy lifestyles. This process is systemically moderated by China’s urban–rural binary structure, gender roles, and class differentiation, suggesting a multi-level, multi-path cumulative mechanism underlying the formation of health inequalities. Policies should promote individual participation in physical activity, enhance health awareness, and advance health equity by addressing family capital and intergenerational educational transmission.

## 1 Introduction

Regular physical activity effectively prevents non-communicable diseases and promotes mental health; exercise is regarded as a key pathway for improving health quality and preventing disease. In China, the widespread insufficiency of physical activity among young and middle-aged adults has become a major contributor to the continued rise in chronic conditions such as obesity, thereby necessitating an in-depth investigation of its social determinants. Existing research has largely demonstrated the beneficial associations of physical exercise on health from physiological and psychological perspectives, yet it has overlooked that exercise is a socialized bodily behavior choice. Beyond individual preference, determinants of exercise behavior include the combined influence of family capital and intergenerational transmission of education. Family capital—a composite concept encompassing economic, cultural and social resources—has long been regarded as a key predictor of offspring developmental outcomes (1). Families with different endowments of capital transmit values, behavioral patterns and resources to their children through intergenerational interaction, thereby shaping life choices and developmental trajectories (2). In the domain of physical activity, prior studies have shown that family economic capital, such as the ability to purchase fitness equipment or pay for sports classes (3, 4); cultural capital, such as parents’ own exercise behaviors and habits (5, 6); and social capital, such as sport-related social networks and community sports resources accessed through family connections (7, 8), all exert positive influences on children’s participation in physical activity. Parents with higher educational attainment typically possess more extensive health knowledge and place greater value on long-term benefits (9). Parental education is positively associated with offspring participation in physical exercise, promoting physical activity through transmission of health cognitions, resource allocation and the creation of a supportive family environment (10). Intergenerational educational transmission may therefore serve as a mediating link between family capital and offspring physical activity.

However, existing studies have predominantly focused on the unidimensional direct relationship between family capital and physical exercise behavior, without examining it from a multidimensional perspective of intergenerational educational transmission. More importantly, the mediating mechanisms through which family capital influences physical exercise behavior have not been adequately demonstrated. In addition, empirical research in this field exhibits clear methodological limitations. Regarding sample selection, many studies rely on regional or non-nationally representative data, which constrains the generalizability of their conclusions; regarding statistical analysis, exercise frequency—commonly measured as an ordered categorical variable—has often been inappropriately modeled using ordinary least squares methods intended for continuous outcomes, potentially yielding biased parameter estimates.

This study uses nationally representative data from the 2023 China General Social Survey and employs an ordered probit model appropriate for ordered dependent variables to systematically investigate the relationships among family capital, intergenerational educational transmission, and physical exercise. Theoretically, by constructing and testing a mediation model of “family capital → intergenerational educational transmission → physical exercise,” the study deepens understanding of the intrinsic mechanisms of intergenerational transmission of health-related behaviors. Practically, the findings can provide empirical support for designing more targeted public health interventions.

## 2 Literature review

### 2.1 The multidimensional influence of family capital on physical exercise

Physical exercise, as a core component of a healthy lifestyle, is profoundly influenced by family capital. The economic, cultural, and social dimensions encompassed by family capital constitute key variables affecting individual exercise behavior (11), each exerting distinct impacts on personal participation in physical activity.

From the perspective of economic capital, families with better financial resources are more able to purchase sports equipment for their children and pay for extracurricular sports training, thereby increasing the frequency and quality of their children’s physical exercise (12). Even within rural areas, differences in family capital exacerbate inequalities in opportunities for physical activity: rural families with greater economic means can offset local resource shortages by obtaining cross-regional resources and traveling to cities for sports training (13). This phenomenon also occurs among urban families, and the supportive role of economic capital strengthens as sports education becomes more professionalized. Regarding the types of sports activities selected, children from urban families are more likely to participate in recreational and skill-based sports, whereas rural children’s physical activities are more often related to productive labor and basic physical conditioning (14). It is noteworthy that economic capital also supports the stability of sports participation: households with private health insurance are more inclined to plan for long-term sports engagement for their children, and this association is more pronounced in families without chronic illnesses (15).

Cultural capital, through the gradual development of behavioral habits and parents’ verbal instruction and role modeling, helps children obtain better educational opportunities (16). Embodied cultural capital—such as parents’ own exercise habits—directly shapes children’s sports values. Children whose parents maintain regular exercise routines are significantly more likely to develop stable patterns of physical activity (17, 18). Objectified cultural capital, including sports books and equipment within the household, provides material and environmental support for children’s participation in physical exercise (19). Moreover, parents’ educational attainment, as an important form of institutionalized cultural capital, indirectly influences children’s sports participation by shaping family educational decisions; highly educated parents place greater value on the physical and mental health benefits of exercise and are better able to guide children in exercising scientifically (20). This transmission effect of cultural capital is also observed across cultural contexts such as Brazil, where higher-education families are more inclined to participate in organized sports activities (21).

The influence of social capital on physical exercise is context-dependent. In urban families, parents’ social networks can help children access high-quality sports education resources; in rural areas, family social capital more often enhances children’s opportunities for physical activity indirectly through neighborly mutual aid and community sports organization. Where regional sports policies promote equalization of public sports resources, the marginal effect of social capital may be partially attenuated (22). Research confirms that the impact of social class on physical exercise strengthens over time: with each upward class step, the probability of engaging in physical exercise increases by 3.5 percentage points, and this effect is more pronounced among urban families (23). In summary, we propose the following research hypotheses:

*H*1a: Family economic capital is positively and significantly associated with offspring’s participation in physical exercise.

*H*1b: Family cultural capital is positively and significantly associated with offspring’s participation in physical exercise.

*H*1c: Family social capital is positively and significantly associated with offspring’s participation in physical exercise.

### 2.2 Intergenerational transmission of education and physical exercise

Intergenerational transmission refers to the extent to which an individual’s behaviors and characteristics recur in subsequent generations (24). Education both facilitates upward mobility in processes of intergenerational social mobility and contributes to the reproduction of social strata across generations (25). The intergenerational transmission of education is a key pathway through which family capital perpetuates advantages across generations; its core mechanisms encompass both resource investment and cultural transmission. In terms of resource investment, the higher parents’ educational attainment, the greater their economic investment in their children’s education, which in turn enhances children’s educational attainment (26, 27). In terms of cultural transmission, parents’ educational values and learning habits are conveyed to their children through daily interactions, constituting an “intergenerational cultural inheritance” whose impact on children’s educational outcomes can even exceed that of economic investment.

Improvements in offspring’s educational attainment further enhance individuals’ sports-related cognitive capacities and ability to access resources, manifested in better understanding of exercise’s health benefits and in recognition of high-quality sports resources, ultimately promoting participation in physical activity (28). Through the cognitive pathway, parents with higher educational attainment are more likely to accurately apprehend the health value of exercise; in families where parents have received higher education, children more readily develop the cognition that “exercise equals health” rather than viewing sport solely as recreation or competition (29). Through the resource pathway, highly educated parents tend to possess broader social networks and stronger capacities to obtain resources, enabling them to secure high-quality sports education resources for their children; this resource advantage is further consolidated via the intergenerational transmission of educational achievement.

Compared with paternal education, maternal education exerts a greater influence on offspring’s attainment of higher education (30). Women with higher education are more likely to break free from traditional gender-role constraints and to engage in various forms of physical activity, a breakthrough often rooted in the intergenerational transmission of family cultural capital, such as parents’ gender-equality beliefs. Along the urban–rural dimension, the intergenerational transmission of education in urban families has a more pronounced effect on physical activity, whereas children from rural families who achieve higher educational levels through intergenerational transmission may still find it difficult to participate fully in physical exercise due to scarcity of public sports resources (22).

The broader social context further modulates the strength of intergenerational transmission of educational advantage. Middle-class parents are better able to mobilize economic, social, and cultural capital to cope with pandemic-related challenges such as online learning, and these individualized strategies exacerbate educational inequality (31). In particular, middle-class parents are increasingly motivated to secure competitive advantage for their children by providing high-quality education and deliberately cultivating skills needed in childhood so that their children can outperform others in educational and social settings (32). In summary, we propose the following research hypotheses:

*H*2a: Family capital is positively and significantly associated with the offspring’s level of education.

*H*2b: Controlling for family capital, the offspring’s educational attainment is positively and significantly associated with their participation in physical exercise.

*H*2c: After including intergenerational transmission of education, the direct associations of economic capital and cultural capital on physical exercise are substantially reduced, while the direct association of social capital becomes non-significant.

## 3 Materials and methods

### 3.1 Data source and sample processing

The data for this study are drawn from the 2023 China General Social Survey (CGSS). The CGSS employs a multistage stratified probability sampling design and covers all 31 provinces, autonomous regions, and municipalities directly under the central government, making it one of the most representative and authoritative databases for studying individual behavior and social stratification in the Chinese context. To ensure sample homogeneity and analytic validity, respondents aged 18 to 65 were retained in accordance with the World Health Organization’s age classification for young and middle-aged adults. Observations with missing or anomalous values on key variables were excluded, yielding a final analysis sample of 2,394 valid cases. All analyses were conducted using Stata 18.0.

### 3.2 Variable definition and measurement

#### 3.2.1 Dependent variable: frequency of physical exercise

The dependent variable is the individual’s frequency of participation in physical exercise. Based on the original response options to questionnaire item “a3009,” we constructed a five-category ordered variable: 1 = “Never,” 2 = “A few times a year or less,” 3 = “A few times a month,” 4 = “A few times a week,” 5 = “Daily.” This measurement aligns with WHO recommendations on regular physical activity and effectively captures variation in the intensity of individuals’ exercise behavior.

#### 3.2.2 Independent variable: family capital

Family capital is operationalized along three dimensions. First, economic capital is measured by the natural logarithm of household annual total income (variable a8a; log_income). Log transformation mitigates right skewness and better reflects marginal effects of income. Second, cultural capital is measured by parents’ highest years of schooling (edu_parents). Fathers’ (a89b) and mothers’ (a90b) educational categories were converted to corresponding years of schooling (for example, “primary school” = 6 years, “junior middle school” = 9 years, “bachelor’s degree” = 16 years, etc.), and the higher of the two values was taken as the indicator. Third, we identified several variables that reflect contemporary social networks (e.g., frequency of socializing, neighborhood interactions, trust), but using them as proxy measures would introduce substantial endogeneity bias. Therefore, this study uses paternal occupational status as the primary proxy for social capital and explicitly assesses. The original occupational classification (variable a89g) was recoded into a four-category ordered variable: 1 = “individual laborer,” 2 = “enterprise/firm,” 3 = “public institution,” 4 = “party and government organs.”

#### 3.2.3 Mediating variable: intergenerational transmission of education

Intergenerational educational transmission is measured by offspring’s educational attainment (edu_offspring). Based on questionnaire item “a7a,” this was coded as a four-category ordered variable: 1 = “primary school or below,” 2 = “junior middle school,” 3 = “senior high school/technical secondary school,” 4 = “junior college or above.” This variable reflects both the influence of parental education on offspring (the outcome of intergenerational transmission) and the offspring’s own educational achievement.

#### 3.2.4 Control variables

To control for other factors that might confound the relationship between family capital and physical exercise, the models include a set of control variables: respondent’s age (age) and its squared term (age2, to capture potential nonlinear lifecycle effects), gender (male, male = 1), hukou urban/rural status (urban, urban = 1), marital status (married, married = 1), and employment status (employed, employed = 1). Additionally, for heterogeneity analyses, an income grouping variable (income_group: 1 = low income, 2 = middle income, 3 = high income) was generated based on tertiles of household income in the sample.

### 3.3 Model specification

#### 3.3.1 Regression analysis model

Given that the dependent variable “frequency of physical exercise” is an ordinal categorical variable, using ordinary least squares may produce biased estimates. Therefore, this study adopts the ordered Probit model as the primary analytical framework. Let $PA_{i}^{*}$ denote the unobserved propensity to engage in physical exercise, and $PA_{i}$ the observed frequency of physical exercise (1 = never to 5 = daily). The ordered probit model assumes that $PA_{i}$ is determined by $PA_{i}^{*}$ via threshold parameters $\mu_{1}<\mu_{2}<\mu_{3}<\mu_{4}$ , such that when $PA_{i}^{*}\leq \mu_{1}$ , $PA_{i}=1$ , when $\mu_{1}<PA_{i}^{*}\leq \mu_{2}$ , $PA_{i}=2$ , and so on.


$$\begin{array}{c} PA_{i}^{*}=\beta_{0}+\beta_{1}\text{EconCapita}l_{i}+\beta_{2}\text{CulturalCapita}l_{i}\\ +\beta_{3}\text{SocialCapita}l_{i}+\gamma X_{i}+\varepsilon_{i} \end{array}$$

EconCapital denotes economic capital, CulturalCapital denotes cultural capital, SocialCapital denotes social capital, and $X_{i}$ is a vector of control variables including age (age), age squared (age^2^), marital status (married), employment status (employed), urban/rural residence (urban), and gender (male); $\varepsilon_{i}$ is the random error term, assumed to follow a standard normal distribution.

#### 3.3.2 Mediation analysis model

To examine the mediating role of offspring education in the relationship between family capital and physical exercise, a recursive modeling framework was constructed. Unlike the traditional stepwise regression approach, this study employed the bias-corrected nonparametric percentile bootstrap method to test the mediation association (33), with 500 resamples. This method directly estimates the confidence interval of the indirect association without relying on asymptotic normality, and offers greater statistical power (34). The mediation model consists of the following two equations:

Step 1:


$$\begin{array}{c} \text{EduOffsprin}g_{i}=\alpha_{0}+\alpha_{1}\text{EconCapita}l_{i}+\alpha_{2}\text{CulturalCapita}l_{i}\\ +\alpha_{3}\text{SocialCapita}l_{i}+\delta X_{i}+u_{i} \end{array}$$

Step 2:


$$\begin{array}{c} PA_{i}^{*}=\theta_{0}+\theta_{1}\text{EconCapita}l_{i}+\theta_{2}\text{CulturalCapita}l_{i}+\theta_{3}\text{SocialCapita}l_{i}\\ +\theta_{4}\text{EduOffsprin}g_{i}+\lambda X_{i}+\nu_{i} \end{array}$$

First estimate coefficient ${\hat{\alpha }}_{1},{\hat{\alpha }}_{2},{\hat{\alpha }}_{3}$ of parental capital on offspring education in the first-step regression; then estimate coefficient ${\hat{\theta }}_{4}$ of offspring education on physical exercise in the second-step regression while controlling for parental capital; next compute the indirect association $\hat{\alpha }\times {\hat{\theta }}_{4}$ . Use bootstrap resampling to obtain the empirical distribution of the indirect effect and construct a 95% confidence interval; if the confidence interval does not include 0, the indirect association is significant, indicating that offspring education mediates the association. Finally, report the direct association with its confidence interval, and calculate the proportion mediated by the indirect association to fully reflect the magnitude of the mediation.

## 4 Research findings

### 4.1 Descriptive statistics

Descriptive statistics indicate that the mean frequency of physical exercise is 2.63, positioned between “several times per month” and “several times per week,” while a relatively large standard deviation suggests substantial heterogeneity in exercise behavior across individuals. The offspring’s educational attainment has a mean of 2.52, corresponding to a level between junior high and senior high school, reflecting a moderate overall educational level in the sample. Regarding family capital, the standard deviation of economic capital is 1.71, indicating pronounced income disparities; cultural capital averages 6.31 years, roughly equivalent to primary school completion, with considerable variation that reflects intergenerational inequality in educational capital; social capital has a mean of 1.40, with the vast majority concentrated among individual laborers and enterprise employees and a very low proportion in party and government agencies. This pattern not only mirrors the prevalence of flexible employment in China’s labor market but also underscores the particularity of using occupational status as a measure of social capital in the Chinese context (Table 1).

**Table 1:** Descriptive statistics of key variables.

Variable	Observations	Mean	SD	Min	Max	Description
Physical activity frequency	2,394	2.63	1.52	1	5	1 = Never, 2 = A few times a year, 3 = A few times a month, 4 = A few times a week, 5 = Daily
Education of offspring	2,394	2.52	1.11	1	4	1 = Primary school or below, 2 = Junior high school, 3 = High school or vocational school, 4 = College or above
Log household income	2,394	10.60	1.71	3.00	16.12	Logarithm of annual income
Parental education years	2,394	6.31	4.48	0	19	Highest number of education years between parents
Father’s occupational status	2,394	1.40	0.76	1	4	1 = Self-employed, 2 = Enterprise, 3 = Public institution, 4 = Government agency
Age	2,394	44.52	11.10	18	60	Age of the respondent (years)
Married	2,394	0.74	0.44	0	1	1 = Married, 0 = Otherwise
Employed	2,394	0.05	0.22	0	1	1 = Employed, 0 = Otherwise
Urban residence	2,394	0.39	0.49	0	1	1 = Urban, 0 = Rural
Male	2,394	0.46	0.50	0	1	1 = Male, 0 = Female

### 4.2 Regression analysis

We systematically examined the statistical associations between household capital in economic, cultural, and social dimensions on frequency of physical exercise using ordered Probit regression. In Model 1, which includes only economic capital, the coefficient is 0.068 (*p* < 0.01), indicating that a 1% increase in household income is associated with a significant increase in exercise frequency. After adding cultural capital in Model 2, the economic capital coefficient slightly declines to 0.063 (*p* < 0.01), while the cultural capital coefficient is 0.037 (*p* < 0.01); both coefficients remained significant and the association for cultural capital was robust. These coefficients are not only highly significant statistically but also substantively meaningful, aligning closely with Bourdieu’s notion of habitus formation (35). This body-mediated “cultural habitus” internalizes physical exercise from an externally incentivized “consumption behavior” into an almost instinctive “way of life,” reflecting the propensity of cultural capital, accumulated through socialization, to guide individuals’ daily practices (36). Consequently, the pathway of cultural capital emphasizes the cultivation of demand and formation of preferences, and—together with economic capital—is linked to sports participation behavior and to improvements in individuals’ educational and socioeconomic outcomes (37), In Model 3, after including social capital, the coefficient is 0.028 (*p* > 0.05) and not statistically significant, suggesting that occupationally based measures of social capital do not exert an independent direct association with physical exercise; conventional occupational classifications may not accurately capture the actual distribution of social capital (Table 2).

**Table 2:** Regression analysis.

Variable	Model 1	Model 2	Model 3
Family capital			
Economic capital (log_income)	0.068*** (0.014)	0.063*** (0.014)	0.062*** (0.014)
Cultural capital (edu_parents)		0.037*** (0.006)	0.035*** (0.006)
Social capital (occ_father)			0.028 (0.034)
Control variables			
Age	−0.053*** (0.016)	−0.052*** (0.016)	−0.050*** (0.017)
Age^2^	0.001*** (0.000)	0.001*** (0.000)	0.001*** (0.000)
Married	0.002 (0.058)	0.005 (0.058)	0.004 (0.058)
Employed	−0.038 (0.105)	−0.011 (0.105)	−0.011 (0.105)
Urban	0.649*** (0.047)	0.569*** (0.048)	0.558*** (0.050)
Male	0.105** (0.045)	0.104** (0.045)	0.102** (0.045)
Model statistics			
Observations	2,394	2,394	2,394
Pseudo R^2^	0.036	0.042	0.042

Standard errors in parentheses; **p < 0.05, ***p < 0.01.

### 4.3 Mediation analysis

Because social capital was not significant in the regression analysis, bias-corrected bootstrap tests with 500 resamples were applied only to economic capital and cultural capital in the mediation analysis. The indirect association of economic capital was 0.027 (95% CI [0.019, 0.035]), and the direct association was 0.042 (95% CI [0.012, 0.069]); the confidence interval for the indirect association did not include zero, and the mediation proportion was 39.2%, indicating that nearly 40% of the association of economic capital on physical exercise is transmitted via offspring’s education. The indirect association of cultural capital was 0.020 (95% CI [0.016, 0.025]), and the direct association was 0.018 (95% CI [0.008, 0.030]); the mediation proportion reached 52.2%, higher than that of economic capital. This reflects different logics of intergenerational transmission for the two forms of capital: cultural capital is inherently transmitted intergenerationally through the educational system, whereas the influence of economic capital follows more diverse pathways. Both indirect associations were significant, confirming that offspring’s education partially mediates the relationship between family capital and physical exercise (Table 3).

**Table 3:** Mediation analysis.

Path	Indirect association	95% confidence interval	Direct association	95% confidence interval	Proportion mediated
Economic capital → Offspring education → Physical activity	0.027***	[0.019, 0.035]	0.042***	[0.012, 0.069]	39.2%
Cultural capital → Offspring education → Physical activity	0.020***	[0.016, 0.025]	0.018***	[0.008, 0.030]	52.2%

Bias-corrected percentile bootstrap with 500 replications; ***p < 0.01; indirect association is a × b, where a = association of family capital on offspring education, b = association of offspring education on physical activity controlling for family capital.

### 4.4 Heterogeneity analysis

Table 4 presents regression results stratified by urban/rural residence, gender, and income groups, along with Suest tests for coefficient differences. In the urban/rural subsamples, the coefficient of economic capital shows a statistically significant association in rural areas (0.053, *p* < 0.05) but not in urban areas; the difference between coefficients is significant (χ^2^ = 4.24, *p* = 0.039), which is consistent with a stronger marginal association in rural contexts. Cultural capital does not show a statistically significant difference between rural and urban samples. Offspring education shows a significant association in both rural (0.332, *p* < 0.05) and urban (0.199, *p* < 0.05) subsamples, with a significant difference between them (χ^2^ = 4.62, *p* = 0.032), suggesting a stronger mediating role of education in rural areas. In the gender-stratified analysis, economic capital shows a significant association for both males and females, with no significant difference between the two. Cultural capital is significantly associated with physical exercise for females (0.024, *p* < 0.05) but not for males; although the difference is not statistically significant, the coefficient is larger for females. In the income subgroup analysis, economic capital shows a significant association in the low-income group (0.206, *p* < 0.05) but not in the high-income group, and the difference between coefficients is highly significant (χ^2^ = 17.53, *p* = 0.000), which corroborates the pattern of diminishing marginal returns of economic capital.

**Table 4:** Heterogeneity analysis by urban–rural, gender, and income groups.

Variable	(1) Urban–rural grouping		(2) Gender grouping		(3) Income grouping		
	Urban	Rural	Male	Female	Low	Middle	High
Family capital							
Log income	−0.017 (0.029)	0.053* (0.016)	0.049 (0.022)	0.039 (0.019)	0.206* (0.043)	0.060 (0.170)	−0.034 (0.029)
Parental education	0.010 (0.010)	0.024* (0.009)	0.009 (0.010)	0.024* (0.009)	0.024 (0.009)	0.008 (0.011)	0.011 (0.014)
Father’s occupation	0.023 (0.045)	0.025 (0.056)	0.024 (0.049)	0.005 (0.048)	0.000 (0.055)	0.051 (0.059)	0.023 (0.068)
Mediating variable							
Education of offspring	0.199* (0.044)	0.332* (0.039)	0.276* (0.043)	0.287* (0.039)	0.305* (0.046)	0.232* (0.050)	0.283* (0.063)
Control variables	Yes	Yes	Yes	Yes	Yes	Yes	Yes
Coefficient difference tests (Suest)							
Log income	urban vs. rural	χ^2^ = 4.24, *p* = 0.039	male vs. female	χ^2^ = 0.10, *p* = 0.750	low vs. high		χ^2^ = 17.53, *p* = 0.000
Parental education	urban vs. rural	χ^2^ = 1.15, *p* = 0.284	male vs. female	χ^2^ = 1.32, *p* = 0.250	low vs. high		χ^2^ = 0.52, *p* = 0.469
Offspring education	urban vs. rural	χ^2^ = 4.62, *p* = 0.032	male vs. female	χ^2^ = 0.04, *p* = 0.848	low vs. high		χ^2^ = 0.08, *p* = 0.782
Model statistics							
Observations	936	1,458	1,101	1,293	1,133	734	527
Pseudo R^2^	0.017	0.041	0.057	0.055	0.064	0.046	0.044

Standard errors in parentheses; *p < 0.05, **p < 0.01, ***p < 0.001.

### 4.5 Robustness checks

We tested the robustness of the core findings using three alternative strategies. First, an OLS model treated the ordered dependent variable as continuous for linear regression; economic capital (0.044, *p* < 0.05) and cultural capital (0.023, *p* < 0.01) showed statistically significant associations, and the coefficient for offspring’s education (0.353, *p* < 0.01) was robust. Second, a Logit model dichotomizing the dependent variable (whether one exercises regularly) showed stronger associations for cultural capital (0.038, *p* < 0.01) and offspring’s education (0.381, *p* < 0.01), indicating a threshold effect of education on establishing regular exercise. Third, after removing the low-variance control variable “employed,” the coefficients for economic capital (0.063, *p* < 0.01) and cultural capital (0.035, *p* < 0.01) were highly consistent with those in the main-effect models. All three robustness checks are consistent with the conclusion that economic and cultural capital show significant positive associations with physical exercise, the mediating role of offspring’s education is stable, and social capital remains non-significant. The core conclusions are not depend on a specific model specification and demonstrate good robustness (Table 5).

**Table 5:** Robustness checks.

Variable	OLS model	Logit model	Model without employed
Economic capital	0.044** (0.018)	0.022 (0.029)	0.063*** (0.014)
Cultural capital	0.023*** (0.008)	0.038*** (0.013)	0.035*** (0.006)
Social capital	0.020 (0.044)	−0.036 (0.067)	0.028 (0.034)
Offspring education	0.353*** (0.036)	0.381*** (0.058)	—
Control variables	Yes	Yes	Yes
Observations	2,394	2,394	2,394
R^2^ / Pseudo R^2^	0.153	0.071	0.042

Standard errors in parentheses; **p < 0.05, ***p < 0.01; OLS treats physical activity as continuous; Logit uses binary outcome (active ≥4); “Model without Employed” excludes the low-variation control variable.

## 5 Discussion

China is currently at a pivotal stage of social transformation, and promoting health equity through participation in physical exercise has become an important element in meeting the public’s aspirations for a better life. Behavioral improvements in physical exercise constitute proactive actions that are associated with multiple factors, including economic and cultural capital and intergenerational educational transmission. Accordingly, the policy implications of this study are discussed in the following three aspects:

First, accurately delineate the distinct pathways through which different forms of capital operate, and implement targeted policies to promote participation in physical exercise. Various forms of familial capital show heterogeneous associations with individual exercise behaviors, consistent with Bourdieu’s theory of capital: different types of capital do not act homogeneously but generate differentiated mechanisms of influence according to their intrinsic properties (2). Economic capital addresses the question of whether material conditions exist; its association are particularly pronounced in rural and low-income populations, suggesting that when basic material resources are lacking, economic capital is the key factor in overcoming “material constraints.” Cultural capital addresses the question of willingness to engage in activity: through prolonged processes of family socialization, health-related values become internalized as individual behavioral norms, potentially transforming physical exercise from a “consumer behavior” into a “way of life” (38). Empirical research in China has demonstrated the role of cultural capital in promoting offspring’s engagement in physical exercise and enhancing their health (39, 40), parental education is significantly and positively associated with college students’ physical activity (41). Although social capital is a primary factor influencing students’ educational choices (42), its direct association in this study is not statistically significant. From a measurement perspective, this study uses only paternal occupational status as the indicator of social capital, which inadequately captures core elements such as social networks, trust, and reciprocity, such measurement error can lead to regression dilution bias, thereby underestimating the true strength of the association of social capital. From a theoretical standpoint, however, social capital is more consequential at critical junctures concerning the ability to access resources. Coleman’s theory of social capital emphasizes that its function lies in facilitating the accumulation of human capital and the acquisition of resources (43). Social relationships centered on “guanxi resources” primarily influence access to resources at critical junctures rather than the shaping of everyday health habits.

Second, leverage the pivotal mediating role of education: intergenerational transmission of education constitutes the core mechanism through which family capital influences offspring’s engagement in physical exercise, and the mediating association manifests a dual-path characteristic. In the antecedent investment stage, economic capital provides material support for offspring to access high-quality education (44), cultural capital shapes offspring’s behavioral dispositions and cognitive capacities (45), and social capital supplies resource support for offspring’s educational choices (46). In the subsequent transformation stage, the health literacy, cognitive skills, and future-oriented time preferences acquired through education prompt offspring to regard physical exercise as a rational, long-term life investment; this process can be construed as an individual’s “cognitive emancipation” (47). It is noteworthy that the mediating share of cultural capital exceeds that of economic capital, which reflects distinct logics in intergenerational transmission: cultural capital inherently requires the education system to achieve intergenerational transmission, whereas the association of economic capital follows more diverse pathways, being deployable both for educational investment and for direct purchase of sports services. Therefore, as a mediator, education exhibits a greater capacity to “absorb” the associations of cultural capital, corroborating the applicability of cultural reproduction theory in the health domain.

Third, attend to group differences and formulate differentiated intervention strategies for distinct populations. Heterogeneity analyses reveal variations in the mechanisms by which family capital operates across groups, reflecting the constraining associations of structural factors on individual agency. Differences in individuals’ exercise behaviors are associated not only with family capital (48) but are also moderated by macro-level social structural factors such as urban–rural residence, gender, and income (49), with family-capital-related factors generally being the dominant force while the moderating roles of macro-structural factors remain non-negligible (50). Urban–rural disparities manifest a divide between “resource constraints” and “cultural orientation.” Income-stratified analyses are consistent with the “law of diminishing marginal returns”: barriers for low-income groups are primarily material, those for middle-income groups may stem from motivation or time-management issues, and barriers for affluent groups arise from time compression associated with high-pressure occupations. Although gender differences did not reach statistical significance, the coefficient for cultural capital is higher in the female subsample, this trend has been corroborated by empirical studies in China: the higher a mother’s level of education, the longer and more frequent her children’s physical activity (51), and mothers with greater educational attainment are more likely to promote children’s exercise by fostering closer family relationships (52), generating a stronger intergenerational transmission effect of cultural capital (32). Promoting participation in physical activity therefore requires context-sensitive approaches: in rural and low-income populations, priority should be given to improving material conditions; in urban and middle-income populations, emphasis should be placed on cultivating healthy habits and cultural norms; and for women, policies should leverage their positive role in transmitting health within families.

This study elucidates the complex relationships among family capital, intergenerational transmission of education, and physical exercise behavior. Although economic capital constitutes the material foundation of family capital and provides the necessary conditions for individual participation in physical activity, cultural capital exert shows a longer-term association with individual behavior (53), with education serving as a central mediating mechanism (54). By adopting a “family capital → educational mediation → health behavior” model, the study extends Bourdieu’s theory of cultural reproduction from the educational domain into the health domain, demonstrating that the formation of individual exercise behavior is not determined by a single factor but results from the joint associations of distinct dimensions of family capital, offspring educational attainment, and broader social structures. The findings reveal differentiated mechanisms of action for different forms of capital and address the applicability of social capital theory within the Chinese context.

As an exploratory study based on national data, this research nevertheless has several limitations. First, the physical activity data used in this study may be subject to social desirability bias, which could lead to overestimation of actual exercise levels. Future research should incorporate objective measurement tools such as accelerometers. Second, the sample in this study was restricted to adults aged 18–60, precluding generalization of the findings to older adults over 60. Future studies should include this population to examine the distinct role of intergenerational family capital on health in later life. Finally, this study’s measurement of social capital is limited to paternal occupational status, which may attenuate estimated coefficients (biasing them toward zero) and thus fail to detect an existing association, rather than indicating that social capital is truly ineffective. Therefore, conclusions regarding the non-significance of social capital should be interpreted with caution. Despite these limitations, the study’s conclusions remain reasonably robust and carry meaningful policy implications regarding the core mechanisms of family capital and educational transmission.

## 6 Conclusion

Physical exercise behavior is not an isolated individual choice but is deeply embedded in the multidimensional capital possessed by families. First, both economic and cultural capital show significant positive associations with participation in physical exercise, but their patterns of association differ fundamentally: economic capital is directly associated with exercise through the provision of resources, whereas cultural capital is associated with health-related dispositions via educational mediation, social capital measured by the father’s occupational status did not show a significant direct association with physical exercise; it is more likely to be indirectly related through pathways of “resource access,” a hypothesis that requires validation in future studies using more comprehensive measurement instruments. Second, intergenerational transmission of education is the key mediating pathway through which family capital is associated with children’s participation in physical exercise; offspring’s educational attainment partially mediates the relationships between both economic and cultural capital and exercise. Finally, the associations of family capital is systematically conditioned by macro-social structures such as urban–rural context, gender, and income.

Future research should further investigate the intergenerational mechanisms underlying health inequities. On the one hand, cross-sectional data limit causal inference; future studies could employ panel data to establish temporal ordering and to better understand the direction of associations. On the other hand, developing multidimensional measures of social capital that better reflect the Chinese context—encompassing core elements such as social networks, trust, and reciprocity—is warranted. Policy efforts should aim to disrupt the intergenerational reproduction of advantages rooted in family capital by reallocating resources to weaken structural constraints and by investing heavily in human capital—particularly by leveraging education to promote liberation of health-related cognition and broad-based empowerment. Through multilevel, systemic interventions, physical activity participation can be increased and health equity advanced.

## Data Availability

Publicly available datasets were analyzed in this study. This data can be found at: Chinese General Social Survey, https://www.cnsda.org/, access requires registration.
