# Supplementary material for: The association between family capital and physical exercise among young and middle-aged adults: the potential mediating pathway of intergenerational transmission of education
Source: Front Public Health. 2026 May 8;14:1791251. doi: 10.3389/fpubh.2026.1791251 (PMC13194150; doi:10.3389/fpubh.2026.1791251)
Supplement: Supplementary file 1 [file Data_Sheet_1.PDF]

Supplementary Material

1.Descriptive. summarize physical_activity edu_offspring log_income edu_parents occ_father age married employed urban male

```
. summarize physical_activity edu_offspring log_income edu_parents occ_father age married employed
rban male

    Variable |        Obs        Mean    Std. dev.       Min        Max
-------------+---------------------------------------------------------
physical_~y |      2,394    2.634503    1.523899          1          5
edu_offspr~g |      2,394    2.522556    1.110916          1          4
 log_income |      2,394    10.59936    1.709284   2.995732    16.1181
 edu_parents |      2,394    6.311195    4.47854          0         19
 occ_father |      2,394    1.399749    .7600668          1          4
-------------+---------------------------------------------------------
         age |      2,394    44.51629    11.10467         18         60
     married |      2,394    .7439432    .4365448          0          1
    employed |      2,394    .0526316    .2233435          0          1
       urban |      2,394    .3909774    .4880713          0          1
        male |      2,394    .4598997    .4984935          0          1
```

2. Main effects model

```
Iteration 0:   log likelihood = -3679.6257
Iteration 1:   log likelihood = -3524.6395
Iteration 2:   log likelihood = -3524.5763
Iteration 3:   log likelihood = -3524.5763

Ordered probit regression                        Number of obs =  2,394
                                                  LR chi2(9)    = 310.10
                                                  Prob > chi2   = 0.0000
Log likelihood = -3524.5763                       Pseudo R2     = 0.0421

--------------------------------------------------------------------------------
physical_activity | Coefficient  Std. err.      z    P>|z|     [95% conf. interval]
------------------+-----------------------------------------------------------------
        log_income |    0.062492    0.013861     4.51   0.000      0.035326   0.089658
       edu_parents |    0.034648    0.006139     5.64   0.000      0.022617   0.046679
        occ_father |    0.027740    0.034024     0.82   0.415     -0.038947   0.094426
              age |  -0.050457    0.016539    -3.05   0.002     -0.082872  -0.018042
             age2 |   0.000601    0.000195     3.09   0.002      0.000219   0.000983
          married |   0.004061    0.057741     0.07   0.944     -0.109110   0.117232
         employed |  -0.010877    0.105139    -0.10   0.918     -0.216945   0.195191
            urban |   0.558205    0.050350    11.09   0.000      0.459520   0.656889
             male |   0.102068    0.045425     2.25   0.025      0.013036   0.191100
------------------+-----------------------------------------------------------------
             /cut1 |  -0.164605    0.354173                      -0.858772   0.529562
             /cut2 |   0.252067    0.354090                      -0.441937   0.946071
             /cut3 |   0.688311    0.353914                      -0.005348   1.381970
             /cut4 |   1.217891    0.354260                       0.523553   1.912228
--------------------------------------------------------------------------------
```

3 Mediation Effect Testing (Bootstrap Method)

3.1 Mediation Effect of Economic Capital

```
. bootstrap r(indirect) r(direct), reps(500) seed(123): boot_mediation_econ

Bootstrap results                               Number of obs =  2,394
                                                Replications  =    500

             |   Observed   Bootstrap                         Normal-based
             | coefficient  std. err.      z    P>|z|     [95% conf. interval]
-------------+----------------------------------------------------------------
       _bs_1 |   0.027016   0.004128     6.54   0.000      0.018925   0.035107
       _bs_2 |   0.042237   0.014518     2.91   0.004      0.013782   0.070692

. estat bootstrap, percentile

Bootstrap results                               Number of obs =  2,394
                                                Replications  =    500

             |    Observed               Bootstrap
             |   coefficient       Bias    std. err.  [95% conf. interval]
-------------+----------------------------------------------------------------
       _bs_1 |   0.02701624   0.0004614   0.00412817   0.0199322   0.0354486 (P)
       _bs_2 |   0.04223676  -0.0004458   0.01451811   0.0120599   0.0692543 (P)
Key: P: Percentile
```

3.2 Mediating Effect of Cultural Capital

```
. bootstrap r(indirect) r(direct), reps(500) seed(123): boot_mediation_cult

Bootstrap results                               Number of obs =  2,394
                                                Replications  =    500

             |   Observed   Bootstrap                         Normal-based
             | coefficient  std. err.      z    P>|z|     [95% conf. interval]
-------------+----------------------------------------------------------------
       _bs_1 |   0.020058   0.002441     8.22   0.000      0.015274   0.024842
       _bs_2 |   0.018390   0.005659     3.25   0.001      0.007299   0.029481

. estat bootstrap, percentile

Bootstrap results                               Number of obs =  2,394
                                                Replications  =    500

             |    Observed               Bootstrap
             |   coefficient       Bias    std. err.  [95% conf. interval]
-------------+----------------------------------------------------------------
       _bs_1 |   0.02005768   0.0001354   0.00244097   0.0156286   0.0252510 (P)
       _bs_2 |   0.01838986   0.0004071   0.00565875   0.0079916   0.0302679 (P)
Key: P: Percentile
```

4. Robustness check

4.1 OLS

```
. regress physical_activity log_income edu_parents occ_father edu_offspring age age2 married empl
d urban male

      Source |       SS           df       MS      Number of obs   =     2,394
-------------+----------------------------------   F(10, 2383)     =     43.05
       Model |  850.296346        10  85.0296346   Prob > F        =    0.0000
    Residual |  4706.89371     2,383  1.97519669   R-squared       =    0.1530
-------------+----------------------------------   Adj R-squared   =    0.1495
       Total |  5557.19006     2,393  2.32226914   Root MSE        =    1.4054


------------------------------------------------------------------------------------
 physical_activity | Coefficient  Std. err.      t    P>|t|     [95% conf. interval]
-------------------+----------------------------------------------------------------
        log_income |   0.043838   0.017909     2.45   0.014      0.008720   0.078956
       edu_parents |   0.022748   0.008188     2.78   0.006      0.006692   0.038804
        occ_father |   0.020487   0.044128     0.46   0.643     -0.066046   0.107019
     edu_offspring |   0.353462   0.036441     9.70   0.000      0.282003   0.424921
               age |  -0.040038   0.021703    -1.84   0.065     -0.082597   0.002521
              age2 |   0.000624   0.000253     2.47   0.014      0.000128   0.001119
           married |  -0.016614   0.074427    -0.22   0.823     -0.162561   0.129334
          employed |   0.030069   0.130349     0.23   0.818     -0.225541   0.285679
             urban |   0.462919   0.071586     6.47   0.000      0.322541   0.603296
              male |   0.135131   0.058203     2.32   0.020      0.020996   0.249264
             _cons |   1.343460   0.478415     2.81   0.005      0.405307   2.281613
```

4.2 Logit

```
. logit active_dummy log_income edu_parents occ_father edu_offspring age age2 married employed urban
male

Iteration 0:   log likelihood = -1523.8149
Iteration 1:   log likelihood = -1416.7448
Iteration 2:   log likelihood = -1415.5491
Iteration 3:   log likelihood = -1415.5484
Iteration 4:   log likelihood = -1415.5484

Logistic regression                                     Number of obs =  2,394
                                                        LR chi2(10)   = 216.53
                                                        Prob > chi2   = 0.0000
Log likelihood = -1415.5484                             Pseudo R2     = 0.0710

-------------------------------------------------------------------------------
   active_dummy | Coefficient  Std. err.      z    P>|z|     [95% conf. interval]
----------------+--------------------------------------------------------------
     log_income |   0.021713   0.028738     0.76   0.450     -0.034612   0.078037
    edu_parents |   0.037597   0.012993     2.89   0.004      0.012131   0.063064
     occ_father |  -0.036319   0.066783    -0.54   0.587     -0.167213   0.094574
  edu_offspring |   0.380623   0.057573     6.61   0.000      0.267782   0.493464
            age |  -0.086375   0.033521    -2.58   0.010     -0.152075  -0.020674
           age2 |   0.001362   0.000391     3.48   0.001      0.000595   0.002129
        married |   0.069715   0.117949     0.59   0.554     -0.161460   0.300891
       employed |   0.071851   0.210014     0.34   0.732     -0.339768   0.483470
          urban |   0.538004   0.109089     4.93   0.000      0.324195   0.751814
           male |   0.208578   0.092029     2.27   0.023      0.028204   0.388951
          _cons |  -1.520528   0.736848    -2.06   0.039     -2.964722  -0.076333
-------------------------------------------------------------------------------
```

4.3 Remove low-variance variables (employed)

```
. oprobit physical_activity log_income edu_parents occ_father age age2 married urban male

Iteration 0:   log likelihood = -3679.6257
Iteration 1:   log likelihood = -3524.6447
Iteration 2:   log likelihood = -3524.5817
Iteration 3:   log likelihood = -3524.5817

Ordered probit regression                         Number of obs =  2,394
                                                   LR chi2(8)    = 310.09
                                                   Prob > chi2   = 0.0000
Log likelihood = -3524.5817                        Pseudo R2     = 0.0421

--------------------------------------------------------------------------------
physical_activity | Coefficient  Std. err.      z    P>|z|     [95% conf. interval]
------------------+-------------------------------------------------------------
        log_income |   0.062566   0.013842     4.52   0.000      0.035436   0.089695
       edu_parents |   0.034673   0.006134     5.65   0.000      0.022651   0.046695
        occ_father |   0.027724   0.034024     0.81   0.415     -0.038962   0.094411
              age | -0.050512   0.016530    -3.06   0.002     -0.082910  -0.018113
             age2 |  0.000601   0.000195     3.09   0.002      0.000220   0.000983
          married |  0.004556   0.057542     0.08   0.937     -0.108225   0.117337
            urban |  0.558478   0.050281    11.11   0.000      0.459928   0.657027
             male |  0.101826   0.045365     2.24   0.025      0.012912   0.190741
------------------+-------------------------------------------------------------
             /cut1 |  -0.164202   0.354148                      -0.858318   0.529915
             /cut2 |   0.252449   0.354067                      -0.441509   0.946407
             /cut3 |   0.688687   0.353891                      -0.004928   1.382301
             /cut4 |   1.218297   0.354234                       0.524010   1.912584
--------------------------------------------------------------------------------
```
